# Corticosteroids and Pediatric Septic Shock Outcomes: A Risk Stratified Analysis

**DOI:** 10.1371/journal.pone.0112702

**Published:** 2014-11-11

**Authors:** Sarah J. Atkinson, Natalie Z. Cvijanovich, Neal J. Thomas, Geoffrey L. Allen, Nick Anas, Michael T. Bigham, Mark Hall, Robert J. Freishtat, Anita Sen, Keith Meyer, Paul A. Checchia, Thomas P. Shanley, Jeffrey Nowak, Michael Quasney, Scott L. Weiss, Sharon Banschbach, Eileen Beckman, Kelli Howard, Erin Frank, Kelli Harmon, Patrick Lahni, Christopher J. Lindsell, Hector R. Wong

**Affiliations:** 1 Division of Critical Care Medicine, Cincinnati Children’s Hospital Medical Center and Cincinnati Children’s Research Foundation, Cincinnati, OH, United States of America; 2 Department of Surgery, University of Cincinnati, Cincinnati, OH, United States of America; 3 UCSF Benioff Children’s Hospital Oakland, Oakland, CA, United States of America; 4 Penn State Hershey Children’s Hospital, Hershey, PA, United States of America; 5 Children’s Mercy Hospital, Kansas City, MO, United States of America; 6 Children’s Hospital of Orange County, Orange, CA, United States of America; 7 Akron Children’s Hospital, Akron, OH, United States of America; 8 Nationwide Children’s Hospital, Columbus, OH, United States of America; 9 Children’s National Medical Center, Washington, DC, United States of America; 10 Morgan Stanley Children’s Hospital, Columbia University Medical Center, New York, NY, United States of America; 11 Miami Children’s Hospital, Miami, FL, United States of America; 12 Texas Children’s Hospital, Houston, TX, United States of America; 13 C. S. Mott Children’s Hospital at the University of Michigan, Ann Arbor, MI, United States of America; 14 Children’s Hospital and Clinics of Minnesota, Minneapolis, MN, United States of America; 15 The Children’s Hospital of Philadelphia, Philadelphia, PA, United States of America; 16 Department of Emergency Medicine, University of Cincinnati College of Medicine, Cincinnati, OH, United States of America; 17 Department of Pediatrics, University of Cincinnati College of Medicine, Cincinnati, OH, United States of America; University of Florida College of Medicine, United States of America

## Abstract

**Background:**

The potential benefits of corticosteroids for septic shock may depend on initial mortality risk.

**Objective:**

We determined associations between corticosteroids and outcomes in children with septic shock who were stratified by initial mortality risk.

**Methods:**

We conducted a retrospective analysis of an ongoing, multi-center pediatric septic shock clinical and biological database. Using a validated biomarker-based stratification tool (PERSEVERE), 496 subjects were stratified into three initial mortality risk strata (low, intermediate, and high). Subjects receiving corticosteroids during the initial 7 days of admission (n = 252) were compared to subjects who did not receive corticosteroids (n = 244). Logistic regression was used to model the effects of corticosteroids on 28-day mortality and complicated course, defined as death within 28 days or persistence of two or more organ failures at 7 days.

**Results:**

Subjects who received corticosteroids had greater organ failure burden, higher illness severity, higher mortality, and a greater requirement for vasoactive medications, compared to subjects who did not receive corticosteroids. PERSEVERE-based mortality risk did not differ between the two groups. For the entire cohort, corticosteroids were associated with increased risk of mortality (OR 2.3, 95% CI 1.3–4.0, p = 0.004) and a complicated course (OR 1.7, 95% CI 1.1–2.5, p = 0.012). Within each PERSEVERE-based stratum, corticosteroid administration was not associated with improved outcomes. Similarly, corticosteroid administration was not associated with improved outcomes among patients with no comorbidities, nor in groups of patients stratified by PRISM.

**Conclusions:**

Risk stratified analysis failed to demonstrate any benefit from corticosteroids in this pediatric septic shock cohort.

## Introduction

The controversy surrounding corticosteroid use in septic shock has yielded multiple adult randomized controlled trials, yet their results are conflicting and a consensus has yet to be reached [1]–[6]. The Surviving Sepsis Campaign guidelines recommend considering corticosteroid usage in patients with refractory shock, defined as those who continue to require vasopressors despite adequate fluid resuscitation [7]. However, physician practices surrounding adjunctive corticosteroid administration vary significantly [8], [9]. Practitioners must weigh the potential hemodynamic improvements seen with corticosteroids against the risks of diminished wound healing, gastrointestinal bleeding, hyperglycemia, and immune suppression [4], [6], [10].

Studies examining corticosteroid use in pediatric septic shock are less abundant than adult studies, but currently the use of corticosteroids is recommended for children with fluid-resuscitated septic shock and evidence of catecholamine resistance or adrenal insufficiency, although these conditions are not evidence- or consensus-defined for children [11]. No large randomized controlled trials examining corticosteroids use in pediatric septic shock have been completed. However, a meta-analysis of small trials showed no benefit attributable to corticosteroids [12]. In addition, two large retrospective observational studies did not show any survival benefit with corticosteroid use in children with septic shock [13], [14]. One of these studies normalized for illness severity and organ failure burden, but did not specifically conduct a risk-stratified analysis [14]. Consequently, the lack of any survival benefit from corticosteroids may be a reflection of the fact that those children who received corticosteroids had a higher initial mortality risk than those who did not receive corticosteroids.

The Surviving Sepsis Campaign guidelines report on an analysis of “low risk” (placebo mortality rate <50%) and “high risk” (placebo mortality >60%) patients, demonstrating no benefit in low risk patients receiving corticosteroids, but a trend toward lower mortality in high risk patients receiving corticosteroids [15]. A recent retrospective, multicenter, propensity-matched study stratified patients with septic shock using the Acute Physiology and Chronic Health Evaluation II (APACHE II) score and reported that corticosteroids were associated with a survival benefit in patients occupying the upper quartile of APACHE II scores for that cohort [16]. Accordingly, the potential benefit of adjunctive corticosteroids for septic shock would be better understood in the context of mortality risk stratification.

Recently, a biomarker-based stratification tool was derived and validated for children with septic shock (Pediatric Sepsis Biomarker Risk Model, PERSEVERE), allowing for stratification into mortality risk categories upon pediatric intensive care unit (PICU) admission [17]–[21]. A potential application of PERSEVERE is to enable stratified analysis of clinical data [22]. We explored the association between corticosteroids and outcomes in children with septic shock stratified by risk of mortality. We hypothesized that, similar to previous reports in adults with septic shock, any potential benefit of corticosteroids in pediatric septic shock is dependent on the initial mortality risk as determined by PERSEVERE. We secondarily hypothesized that the potential benefit of corticosteroids would also be dependent on illness severity as determined by the Pediatric Risk of Mortality (PRISM) score.

## Materials and Methods

### Ethics statement

All study subjects were enrolled after written informed consent from parents or legal guardians. The Institutional Review Boards (IRB) of each participating institution approved secondary use of biological specimens and clinical data: Cincinnati Children’s Hospital Medical Center, UCSF Benioff Children’s Hospital Oakland, Penn State Hershey Children’s Hospital, Children’s Mercy Hospital, Children’s Hospital of Orange County, Akron Children’s Hospital, Nationwide Children’s Hospital, Children’s National Medical Center, Morgan Stanley Children’s Hospital, Columbia University Medical Center, Miami Children’s Hospital, Texas Children’s Hospital, CS Mott Children’s Hospital at the University of Michigan, Children’s Hospital and Clinics of Minnesota, and The Children’s Hospital of Philadelphia.

### Study and Data Collection

The patient cohort (n = 496) was derived from an ongoing multicenter pediatric septic shock database, which has been previously described in detail [23]–[25]. Briefly, children admitted to the PICU meeting pediatric-specific criteria for septic shock were eligible for enrollment [26]. After informed consent from parents or legal guardians, blood samples were obtained as close to the time of meeting criteria for septic shock as possible (<24 hours). Clinical and laboratory data were collected through the first seven days of PICU admission. Mortality was tracked for 28 days after enrollment and organ failure was defined using pediatric-specific criteria [26]. Any neonates (subjects less than 28 days of age) included in the study were full term and admitted to the PICU with septic shock following discharge to home after birth. Subjects were enrolled between May 2002 and May 2013.

### Patient Classification and Stratification

We surveyed the medication fields of the clinical database to determine if the study subjects received systemic corticosteroids. Subjects receiving any formulation of systemic corticosteroids during the first 7 days of meeting criteria for septic shock were classified in the corticosteroid group. The one exception was subjects who received dexamethasone for less than 48 hours for airway edema. These patients were classified in the no corticosteroid group (13 subjects). All other subjects were classified in the no corticosteroid group. We were unable to consistently determine dosages or the clinical indications for corticosteroids in all subjects.

The blood sample was used to stratify subjects into mortality risk strata using PERSEVERE [18], [19]. Based on a histogram showing three main bins, we stratified subjects into low, intermediate, or high risk categories. The “low risk” population was defined as those subjects with a mortality probability ≤2.5%. The “intermediate risk” group was defined as those subjects with a mortality probability >2.5% up to and including 26.7%. The “high risk” group was defined as subjects with a mortality probability >26.7%. All subjects in this study were previously included in the derivation (n = 353) and validation (n = 114) of PERSEVERE.

In a sensitivity analysis, we included only the subjects without comorbidities as an indirect means of selecting only those subjects who received corticosteroids for the indication of refractory shock. In a secondary analysis, subjects were stratified based on the PRISM score tertiles for this cohort. The first tertile contained subjects with PRISM scores ≤10, the second tertile contained subjects with PRISM scores from 11 through 17, and the third tertile contain subjects with PRISM scores >17. We selected this approach to mirror the three PERSEVERE-based risk strata.

### Data Analysis

Statistical analyses were conducted using SigmaStat Software (Systat Software, Inc., San Jose, CA). Initially, data are described using medians, interquartile ranges, frequencies, and percentages. Comparisons between study groups used the Mann-Whitney U-test, Chi-square, or Fisher’s Exact tests, as appropriate.

The association between corticosteroids and outcome was modeled using logistic regression. First, we modeled the probability of all cause 28-day mortality. Second, we modeled the probability of a composite endpoint termed “complicated course”, which is defined as either death within the 28-day study period, or persistence of two or more new organ failures at 7 days after meeting criteria for septic shock, as previously described [20], [22], [27]. Complicated course is not a validated endpoint, but is intended to serve as a pragmatic clinical endpoint that captures both septic shock-related morbidity and mortality.

## Results

### Demographics and clinical characteristics

Among the subjects who received corticosteroids, 78% were prescribed hydrocortisone, 16% were prescribed methylprednisolone, and 6% were prescribed dexamethasone. The median duration of corticosteroid prescription was 5 days (maximum days recorded = 7, interquartile range [IQR], 3–7). The median day of septic shock at which corticosteroids were initially prescribed was day 1 (IQR, 1–1).

Table 1 describes the demographic and clinical characteristics of subjects who received corticosteroids (n = 252) and subjects who did not receive corticosteroids (n = 244). Subjects who received corticosteroids had higher rates of complicated course and mortality, a higher median PRISM score, a higher median number of organ failures, and a greater requirement for vasoactive medications, when compared to subjects who did not receive corticosteroids. A greater proportion of subjects who received corticosteroids had a comorbidity (Table S1). PERSEVERE-based mortality risk did not differ between the two groups. No other differences were noted.

**Table 1:** Clinical and demographic data for the study subjects.

	No Corticosteroids(n = 244)	Corticosteroids(n = 252)	P value
Age in years, median (IQR)	2.8 (0.7–7.1)	3.4 (1.3–7.0)	0.097
Males, N (%)	137 (56)	140 (56)	0.966
Deaths, N (%)	21 (9)	43 (17)	0.005
Complicated course, N (%)	53 (22)	80 (32)	0.016
PRISM score, median (IQR)	11 (7–18)	15 (10–22)	<0.001
Mean days to death ± SD	5.4±6.8	5.4±5.7	0.955
Median days to death (IQR)	3 (1–17)	4 (1–12)	0.553
PERSEVERE-based mortality probability, mean(95% C.I.)	9.8 (7.9–11.7)	12.7 (10.6–14.8)	0.090
Maximum number of organ failures, median (IQR)	2 (1–3)	2 (2–3)	0.025
Gram-positive bacteria, N (%)	60 (25)	58 (23)	0.759
Number of vasoactive agents at the time ofcorticosteroid initiation, median (IQR)	–	2 (1–2)	–
Maximum number of simultaneous vasoactive agentsduring the first 7 days, median (IQR)	1 (1–2)	2 (1–3)	<0.001
Gram-negative bacteria, N (%)	45 (18)	58 (23)	0.252
Other organism, N (%)	19 (8)	25 (10)	0.525
Negative cultures, N (%)	120 (49)	111 (44)	0.291
Comorbidity, N (%)	70 (29)	105 (42)	0.003

### Association between corticosteroids and mortality

Table 2 shows the association between corticosteroids and mortality. In the overall cohort, there were 64 deaths (13%) and the use of corticosteroids was associated with an increased risk of death (OR 2.3, 95% CI 1.3–4.0, p = 0.004). Within each risk stratum, there was no association between the use of corticosteroids and mortality.

**Table 2:** Association between corticosteroids and mortality.

Group	# ofdeaths	Subjects receiving ≥2vasoactive medications # (%)	OR	95% C.I.	P value
All subjects (n = 496)	64	242 (49)	2.304	1.312–4.046	0.004
Low risk subjects (n = 323)	7	132 (41)	6.898	0.821–57.595	0.075
Intermediate risk subjects (n = 117)	27	66 (56)	1.371	0.563–3.343	0.487
High risk subjects (n = 56)	30	44 (79)	2.333	0.780–6.980	0.130

### Association between corticosteroids and complicated course

Table 3 shows the association between corticosteroids and a complicated course. Overall, there were 133 (27%) subjects with a complicated course and the use of corticosteroids was associated with an increased risk of a complicated course (OR 1.7, 95% CI 1.1–2.5, p = 0.012). When stratified into the three PERSEVERE-based mortality risk strata, there was no association between the use of corticosteroids and the risk for a complicated course.

**Table 3:** Association between corticosteroids and complicated course (CC).

Group	# with CC	OR	95% C.I.	P value
All subjects (n = 496)	133	1.676	1.119–2.510	0.012
Low risk subjects (n = 323)	37	1.735	0.865–3.482	0.121
Intermediate risk subjects (n = 117)	54	1.000	0.481–2.078	1.000
High risk subjects (n = 56)	42	2.667	0.773–9.194	0.120

### Analysis of subjects without comorbidities

Since we were unable to consistently determine the clinical indications for corticosteroids in all subjects, we conducted a sensitivity analysis limiting the dataset to patients without comorbidities (n = 321). We reasoned that the majority of these subjects were administered corticosteroids for the indication of refractory shock.

Among the subjects without comorbidities who received steroids, 79% were prescribed hydrocortisone, whereas 15% were prescribed methylprednisolone, and 6% were prescribed dexamethasone. The median duration of steroid prescription was 4 days (maximum days recorded = 7, IQR, 3–7). The median day of septic shock at which corticosteroids were initially prescribed was day 1 (IQR, 1–1).

Table 4 describes subjects without comorbidities grouped by whether or not they received corticosteroids. Subjects who received corticosteroids had higher rates of complicated course and mortality, higher PRISM scores, higher median number of organ failures, and a greater requirement for vasoactive medications, compared to subjects who did not receive corticosteroids. PERSEVERE-based mortality risk did not differ between the two groups. No other differences were noted.

**Table 4:** Clinical and demographic data for the study subjects without comorbidities.

	No Corticosteroids(n = 174)	Corticosteroids(n = 147)	P value
Age in years, median (IQR)	2.4 (0.6–6)	2.2 (1.0–5.5)	0.952
Males, N (%)	92 (52.9)	83 (56.5)	0.595
Deaths, N (%)	14 (8)	27 (18.4)	0.010
Complicated course, N (%)	37 (21.2)	51 (34.7)	0.010
PRISM score, median (IQR)	11 (7–17.3)	16 (11–22)	<0.001
Mean days to death ± SD	5.9±7.7	4.7±4.9	0.540
Median days to death (IQR)	3 (1–6)	3 (2–6)	0.759
PERSEVERE-based mortality probability,mean (95% C.I.)	11.2% (8.7–13.7)	14.8% (11.7–17.9)	0.191
Maximum number of organ failures, median (IQR)	2 (1–3)	2 (2–3)	0.020
Number of vasoactive agents at the time ofcorticosteroid initiation, median (IQR)	–	2 (1–3)	–
Maximum number of simultaneous vasoactiveagents during the first 7 days, median (IQR)	1 (1–2)	2 (1–3)	<0.001
Gram-positive bacteria, N (%)	44 (25.3)	36 (24.7)	1.000
Gram-negative bacteria, N (%)	24 (13.8)	29 (19.9)	0.192
Other organism, N (%)	10 (5.7)	16 (11)	0.135
Negative cultures, N (%)	95 (54.6)	66 (45.2)	0.118

Table 5 shows the association between corticosteroids and mortality in the subjects without comorbidities. There were 41 deaths (13%) and the use of corticosteroids was associated with an overall increased risk of death (OR 2.6, 95% CI 1.3–5.1, p = 0.007). Within mortality risk strata, no benefit of corticosteroids was observed.

**Table 5:** Association between corticosteroids and mortality in subjects without comorbidities.

Group	# of deaths	SubjectsReceiving ≥2vasoactivemedications # (%)	OR	95% C.I.	P value
All subjects (n = 321)	41	162 (50)	2.571	1.293–5.114	0.007
Low risk subjects (n = 202)^1^	1	85 (42)	–	–	–
Intermediate risk subjects (n = 71)	14	40 (56)	1.707	0.524–5.558	0.375
High risk subjects (n = 48)	26	37 (77)	2.700	0.828–8.807	0.100

1 The number of deaths is too small to estimate the odds ratio.

Table 6 shows the associations between corticosteroid use and complicated course among subjects without comorbidities. Within this population, there were 88 subjects (27%) with a complicated course and the use of corticosteroids was associated with an increased risk of a complicated course (OR 2.0, 95% CI 1.2–3.2, p = 0.008). When stratified into the three PERSEVERE-based mortality risk strata, there was no association between corticosteroid use and complicated course.

**Table 6:** Association between corticosteroids and complicated course (CC) in subjects without comorbidities.

Group	# with CC	OR	95% C.I.	P value
All subjects (n = 321)	88	1.967	1.196–3.234	0.008
Low risk subjects (n = 202)	21	2.404	0.949–6.090	0.064
Intermediate risk subjects (n = 71)	30	1.013	0.394–2.604	0.978
High risk subjects (n = 48)	37	3.231	0.795–13.123	0.101

### Association between corticosteroids and outcomes using PRISM-based stratification

Because PRISM scores are more familiar to the critical care medicine field than PERSEVERE, we conducted a secondary regression analysis using subjects grouped into low, medium, and high risk based on PRISM score tertiles. For all subjects, higher PRISM scores were associated with increased risk of mortality (OR 1.09, 95% CI 1.07–1.12, p<0.001) and complicated course (OR 1.08, 95% CI 1.06–1.11, p<0.001). Table 7 shows the association between corticosteroids and mortality within each PRISM-based stratum. There was no association between the use of corticosteroids and mortality within each risk stratum. Similarly, Table 8 shows the association between corticosteroids and a complicated course within each risk stratum. There was no association between the use of corticosteroids and a complicated course within each risk stratum.

**Table 7:** Association between corticosteroids and mortality based on PRISM tertiles.

PRISM Tertile	# ofdeaths	Subjects receiving ≥2 vasoactivemedications # (%)	OR	95% C.I.	P value
1^st^ (n = 172)	7	61 (35)	4.048	0.762–21.494	0.101
2^nd^ (n = 169)	13	89 (53)	1.651	0.477–5.710	0.428
3^rd^ (n = 155)	44	92 (59)	1.633	0.781–3.412	0.192

**Table 8:** Association between corticosteroids and complicated course (CC) based on PRISM tertiles.

PRISM Tertile	# with CC	OR	95% C.I.	P value
1^st^ (n = 172)	20	2.571	0.991–6.671	0.052
2^nd^ (n = 169)	41	0.901	0.444–1.825	0.771
3^rd^ (n = 155)	72	1.355	0.709–2.590	0.358

## Discussion

We examined the association between corticosteroid administration and outcomes in a large, heterogeneous cohort of children with septic shock from multiple institutions across the United States. When including all subjects regardless of initial mortality risk, corticosteroids were associated with poorer outcomes. We note that subjects who received corticosteroids had greater illness severity as measured by PRISM score, mortality, organ failure burden, and requirement for vasoactive medications. Thus, the finding that corticosteroids were associated with poorer outcomes in the overall cohort is likely confounded by illness severity.

To account for this important confounder, we stratified the subjects into three mortality risk strata using PERSEVERE. Within each mortality risk strata, we observed no benefits associated with corticosteroid use. These findings are consistent with previous studies showing no outcome benefit associated with corticosteroid administration in children with septic shock [12]–[14].

Menon et al. conducted a meta-analysis of 447 selected cases and found no survival benefit associated with corticosteroids in children with septic shock [12]. Markovitz et al. studied over 6,000 subjects using the Pediatric Health Information System administrative database and reported that corticosteroids were associated with increased mortality, although they were not able to account for illness severity [13]. Zimmerman et al. analyzed the results of the largest interventional clinical trial in pediatric sepsis and found that corticosteroids were not associated with improved outcomes [14]. The subjects in the study by Zimmerman et al. had similar PRISM scores and organ failure burden in the two treatment groups, but the study did not explicitly stratify for initial mortality risk as done here.

An important limitation of this retrospective study is that the indication for corticosteroids was not standardized across the study subjects, nor was the general clinical care for sepsis. Corticosteroid administration was at the discretion of the attending physician, therefore some corticosteroid administration might have been for indications other than septic shock. We note, however, that 78% of the overall cohort was prescribed hydrocortisone and the median day of hydrocortisone administration was “day 1” of meeting criteria for septic shock. In addition, subjects who received corticosteroids had a greater requirement for vasoactive medications. These observations are consistent with the Surviving Sepsis Campaign recommendations for adjunctive corticosteroid administration in patients with septic shock. To further account for this confounder, we conducted a sensitivity analysis that excluded subjects with comorbidities. By limiting subjects to those without comorbidities, we hoped to exclude subjects who received corticosteroids for chronic diseases and indications other than septic shock. Similar to the overall cohort, the subjects who received corticosteroids in this cohort with no comorbidity had greater illness severity and the use of corticosteroids was associated with worse outcomes overall. When this cohort with no comorbidity was stratified by initial mortality risk using PERSEVERE, there was no benefit associated with corticosteroid use in any risk stratum. The same pattern was observed using the more familiar PRISM score to stratify patients: we found no survival benefit associated with the use of corticosteroids in any PRISM-based risk stratum.

The timing of corticosteroids was not standardized for patients included in this analysis. This may be important because a recent, small study suggests that administration of corticosteroids within nine hours of vasopressor initiation leads to improved outcomes [28]. Conversely, a much larger study by Casserly et al. showed that adjunctive corticosteroids are associated with increased adjusted hospital mortality even when prescribed within eight hours of vasopressor initiation [3]. It is unclear, then, how variable timing of corticosteroids might have influenced our findings.

Another limitation of our study is that we could not determine whether patients were diagnosed with relative adrenal insufficiency. It has been suggested that patients with relative adrenal insufficiency may benefit the most from corticosteroid administration [1]. While relative adrenal insufficiency has been shown to exist in critically ill children depending on the definition used, there is little data on the association between corticosteroids and pediatric outcomes in the context of relative adrenal insufficiency [29]. Therefore, relative adrenal insufficiency may remain a confounder in our study and should be incorporated in future studies. Finally, the retrospective design of our study makes it impossible to determine whether associations between corticosteroid use and poorer outcomes in the overall cohort are causal or simply associated with initial mortality risk. To explore this relationship further, a randomized controlled trial using mortality risk-stratification at study entry is warranted.

In conclusion, risk stratified analysis failed to demonstrate any benefit from corticosteroids in this pediatric septic shock cohort. Thus, apart from children receiving chronic steroids and children with “classic” adrenal insufficiency, the accumulating evidence does not support the routine use of corticosteroids in children with septic shock in the absence of a randomized trial.

## Supporting Information

Table S1 Table of comorbidities.
(DOCX)
