# Supplementary material for: Corticosteroids and Pediatric Septic Shock Outcomes: A Risk Stratified Analysis
Source: PLoS One. 2014 Nov 11;9(11):e112702. doi: 10.1371/journal.pone.0112702 (PMC4227847; doi:10.1371/journal.pone.0112702)
Supplement: Table S1 — Table of comorbidities. (DOCX) [file pone.0112702.s001.docx]

**S1:** Comorbid conditions

| **Comorbidity** | **N** |
| --- | --- |
| Acute lymphoblastic leukemia | 9 |
| Acute lymphoblastic leukemia and stem cell transplantation | 4 |
| Acute myelogenous leukemia | 1 |
| Acute myelogenous leukemia and stem cell transplantation | 1 |
| Aplastic anemia | 3 |
| Aplastic anemia and stem cell transplantation | 1 |
| Astrocytoma | 1 |
| Brain tumor | 2 |
| Burkitt’s Lymphoma and stem cell transplantation | 1 |
| Burn victim | 1 |
| CHARGE Syndrome | 1 |
| Chronic granulomatous disease | 1 |
| Chronic lung disease | 2 |
| Chronic lymphopenia | 1 |
| Congenital heart disease | 14 |
| Cri du chat | 1 |
| Cyclic neutropenia | 1 |
| DiGeorge Syndrome | 2 |
| Down Syndrome | 6 |
| End stage liver disease | 4 |
| End stage renal disease | 8 |
| Epilepsy | 2 |
| Fanconi anemia and stem cell transplantation | 2 |
| Glycogen storage disease | 2 |
| Hemophagocytic lymphohistiocytosis | 5 |
| Hemophagocytic lymphohistiocytosis and stem cell transplantation | 2 |
| Hepatoblastoma | 1 |
| Histiocytosis and stem cell transplantation | 1 |
| Hydrocephalus | 1 |
| IPEX syndrome and stem cell transplantation | 1 |
| Liver transplantation | 5 |
| Medulloblastoma | 4 |
| Meta leukodystrophy and stem cell transplantation | 1 |
| Mitochondrial disorder | 3 |
| Morbid obesity | 1 |
| Multi-visceral transplant | 3 |
| Nephrotic syndrome | 2 |
| Neuroblastoma | 3 |
| Neuroblastoma and stem cell transplantation | 1 |
| Neuro-developmental delay | 28 |
| Neuromuscular disorder | 1 |
| Optic nerve glioma | 1 |
| Pallister Killian Syndrome | 1 |
| Pituitary dwarfism | 1 |
| Prader Willi syndrome | 1 |
| Pulmonary hypertenstion | 1 |
| Renal transplantation | 1 |
| Retinoblastoma and stem cell transplantation | 1 |
| Rhabdomyosarcoma | 2 |
| Severe combined immuno deficiency | 3 |
| Short gut syndrome | 8 |
| Spinal muscular atrophy | 1 |
| Stem cell transplantation | 6 |
| Systemic lupus erythematousus | 1 |
| Tracheal stenosis | 4 |
| Trichothiodystrophy | 1 |
| Trisomy 18 | 2 |
| Type 1 diabetes | 1 |
| Unspecified genetic disorder | 2 |
| Unspecified metabolic disorder | 1 |
| WAGR syndrome | 1 |
| Wiskott Aldrich syndrome | 1 |
